# Guest Editorial

**Published:** 2010-09-15

**Authors:** Navneet Grewal

**Affiliations:** President ISPPD, Professor and Head, Department of Pedodontics Government Dental College Amritsar, Punjab, India

**From the Secretariat of National ISPPD Conference, Amritsar,...**

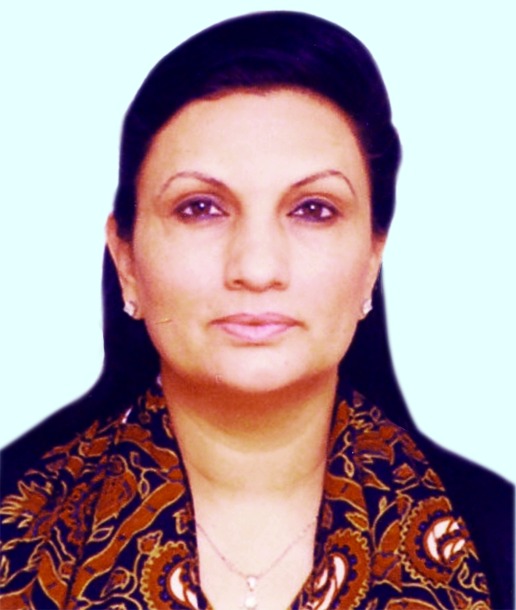



The triumphs and downfalls of the Commonwealth Games have taught us one important lesson that no matter what happens, we Indians have the tremendous ability to rise and shine because of our immense talent and “never say die” spirit.

Perhaps this spirit is also required in the oral health scenario of our country. Today represents the triumphs and downfalls of dentistry in India and that is why we need to share common language based on good judgement and clinical evidence to take oral health status of our society to the pinnacle of excellence. Ours is a treatment-oriented society where people visit a dentist only when there is a dire need to do so. Hence, today calls for a need to emphasize on forming firm oral health foundations in the most dynamic part of our population-CHILDREN. If we do not eliminate the disease burden of society we are all going to pay and so will our children and grandchildren keep on spending alarmingly on oral diseases.

Pediatric dentists have the ability to be the forerunners in forming these foundations by being the prime educators of modern treatment modalities to the parents and children because children are “Gods opinion that the world should go on.” This journal of clinical pediatric dentistry opens up vistas of immense knowledge and research which is of global standards and allows us to perceive children as not “Things to be molded but people to be unfolded.”

**Navneet Grewal**

President ISPPD

Professor and Head

Department of Pedodontics

Government Dental College

Amritsar, Punjab, India

